# Circulating ANGPTL8 Is Associated with the Presence of Metabolic Syndrome and Insulin Resistance in Polycystic Ovary Syndrome Young Women

**DOI:** 10.1155/2019/6321427

**Published:** 2019-06-27

**Authors:** Danlan Pu, Ling Li, Jingxia Yin, Rui Liu, Gangyi Yang, Yong Liao, Qinan Wu

**Affiliations:** ^1^Department of Endocrine Nephropathy, Chongqing University Cancer Hospital and Chongqing Cancer Institute and Chongqing Cancer Hospital, 400030, China; ^2^Key Laboratory of Diagnostic Medicine (Ministry of Education) and Department of Clinical Biochemistry, College of Laboratory Medicine, Chongqing Medical University, 400016, China; ^3^Department of Endocrinology, Armed Police Hospital of Chongqing, Chongqing, China; ^4^Department of Endocrinology, The Second Affiliated Hospital, Chongqing Medical University, Chongqing, China

## Abstract

**Background:**

ANGPTL8 has been reported to be a regulator of lipid metabolism, and it is associated with insulin resistance (IR) and metabolic syndrome (MetS). We investigated whether ANGPTL8 plays a role in MetS.

**Methods:**

ANGPTL8 and adiponectin concentrations were measured in PCOS patients with or without MetS and in their corresponding healthy controls. The association of circulating ANGPTL8 with adiponectin and other parameters was also examined.

**Results:**

Circulating ANGPTL8 concentrations were higher in PCOS women with MetS than in those without MetS and in the controls (*P* < 0.01). ANGPTL8 was positively correlated with age, BMI, FAT%, WHR, SBP, TG, FBG, HbA1c, Fins, and HOMA-IR (all *P* < 0.01) in the study populations and negatively associated with adiponectin and *M*-values (*P* < 0.001). In addition, ANGPTL8 was positively correlated with PRL, LH, TEST, and FAI and negatively correlated with SHBG (all *P* < 0.01). ROC curve analyses showed that the AUC_MetS_ was 0.87 (*P* < 0.001), with a sensitivity of 92.4% and specificity of 75.4%, and the AUC_IR_ was 0.82 (*P* < 0.01), with a sensitivity of 76.4% and specificity of 75.6%.

**Conclusion:**

ANGPTL8 levels progressively decrease from PCOS patients with MetS to those without MetS and may be a serum marker associated with the degree of metabolic disorders.

## 1. Introduction

Metabolic syndrome (MetS) is a cluster of dysmetabolic diseases that increase the risk of cardiovascular disease (CVD), hypertension, and type 2 diabetes mellitus (T2DM) [1, 2]. The working definitions of MetS include abdominal obesity, hyperlipidaemia, hyperglycaemia, and hypertension [3]. MetS subjects have a 5-fold increased risk of T2DM and a 2-fold increased risk of CVD [4, 5]. Atherosclerosis in humans is induced by various components of MetS, and when these components occur together, they are more significant in promoting atherosclerosis [6, 7]. Therefore, in both developed and developing countries, MetS is a fairly serious public health problem [8–10]. It is thus important to improve the preventive and therapeutic strategies of MetS.

Recent studies have reported that some cytokines, such as bone morphogenetic protein-9 (BMP-9) [11], angiopoietin-like protein 8 (ANGPTL8) [12], and irisin [13], are associated with MetS in humans. Therefore, the relationship between these cytokines and the occurrence of MetS has been widely studied. ANGPTL8 is a liver-produced protein that has been found to be related to lipid metabolism, MetS, and insulin resistance (IR) [12, 14–16]. ANGPTL8 expression in adipose tissues and the liver was found to be higher relative to that in other tissues [15, 16]. As a typical member of the ANGTPL family, ANGPTL8 regulates triacylglycerol (TG), high-density lipoprotein cholesterol (HDL-C), and low-density lipoprotein cholesterol (LDL-C) levels by interacting with ANGTPL3 [14, 16–18]. In type 1 diabetes mellitus (T1DM) and insulin-deficient animals, serum ANGPTL8 levels were increased [14, 19]. In T2DM animals, hepatic ANGPTL8 expression was upregulated, suggesting that ANGPTL8 is regulated by IR [19]. Recently, some contrary reports have found that ANGPTL8 is not associated with IR and the proliferation of islet *β* cells [20]. Thus, with regard to the association between ANGPTL8 and IR, the present results are contradictory, and further study is needed.

Polycystic ovary syndrome (PCOS) is a complex endocrine and metabolic disorder, characterized by chronic anovulation and hyperandrogenism. It is well known that insulin resistance play an important role in the pathogenesis of PCOS and Mets. Whether there is any difference between PCOS subjects with Mets and those without Mets in the level of insulin resistance and the ANPTL8 are not known.

More recently, Abu-Farha et al. reported that circulating ANGPTL8 is elevated in MetS individuals and is significantly related to high-sensitivity C-reactive protein (CRP), highlighting its role in dysmetabolism and chronic inflammation [12]. To exclude the effects of age and sex, in the current study, teenage women were employed as study individuals. Our results showed that serum ANGPTL8 levels in MetS women are significantly elevated relative to healthy controls and associated with adiponectin (ADI) levels and IR.

## 2. Materials and Methods

In the current study, 241 teenage women (98 healthy controls and 143 polycystic ovary syndrome (PCOS) subjects) were recruited from the community through advertisement or routine medical check-up in the Department of Endocrinology at the Second Affiliated Hospital of Chongqing Medical University between 2016 and 2017. The diagnosis of PCOS was based on the 2003 Rotterdam consensus (the Rotterdam ESHRE/ASRM-Sponsored PCOS Consensus Workshop Group) [21]. MetS was diagnosed by three or more of the following metabolic risk factors which were clearly defined by the International Diabetes Federation and the American Heart Association in 2009: (1) central obesity (waist circumference (WC) ≥80 cm in females and ≥90 cm in males), (2) hypertriglyceridaemia (triglyceride (TG) ≥1.69 mmol/L), (3) HDL-C <1.29 mmol/L in females and <1.04 mmol/L in males, (4) hyperglycaemia (FBG ≥5.6 mmol/L or T2DM), and (5) hypertension (sitting blood pressure (BP) ≥130/85 mmHg, taken as a mean of two readings obtained after resting for 10-15 minutes or taking oral antihypertensive medication). The exclusion criteria include cancer, cirrhosis, positive infection, heart failure, long-term treatment with steroids, or other medical problems. All patients with MetS were newly diagnosed without any drug treatment. Healthy controls without clinical evidence of major diseases were screened from the community through advertisement or routine medical check-up. These individuals did not take any medicine. This study was conducted according to the Declaration of Helsinki and was supported by the ethics committee of our hospital. Informed consent was obtained from participants, which were given a full explanation of the study. This study was registered with the Chinese Clinical Trial Registry at https://www.chictr.org (CHICTR-OCC-13003185).

### 2.1. Anthropometric and Biochemical Analyses

All participants underwent a physical examination. Anthropometry was performed under standardized conditions by trained staff before breakfast. Body weight and height were examined by a trained nurse, with participants wearing light indoor clothing and nothing on their feet (barefooted), using calibrated portable electronic weighing scales. The body mass index (BMI) was calculated as weight (kg) divided by squared height (metres). WC and hip circumference (HC) were measured by the same nurse and recorded to the nearest 0.1 cm. The waist-to-hip ratio (WHR) was calculated by WC/HC. The BP was measured on the nondominant arm using a mercury sphygmomanometer in all individuals after resting for at least 10 minutes. We used bioelectrical impedance (BIA-101; RJL Systems) to examine the percentage of body fat (FAT%). The homeostasis model assessment of IR (HOMA-IR) was calculated by the following equation: HOMA − IR = fasting insulin (FIns, mU/L) × fasting blood glucose (FBG, mmol/L)/22.5 [22]. Blood samples were collected after fasting for 10-14 h and centrifuged to separate the serum. HbA1c, glucose, insulin, and lipids were measured routine chemistry laboratory at the hospital.

### 2.2. Hormone Measurement

Blood samples were collected in the early follicular phase (days 3 to 5 of the menstrual cycle) in the control group. Blood samples were collected after a spontaneous bleeding episode or upon first examination in PCOS women. Serum hormone concentrations, including luteinizing hormone (LH), follicle-stimulating hormone (FSH), testosterone, and progesterone (Prog), were measured with a well-established electrochemiluminescence immunoassay using COBAS E immunoassay analysers (Roche Diagnostics GmbH). Total testosterone levels were measured with a coated tube radioimmunoassay (RIA; DiaSorin, S. p. A, Saluggia, Italy, and Diagnostic Products Corporation).

Dehydroepiandrosterone sulfate (DHEA-S) and sex hormone-binding globulin (SHBG) were detected using an automated analyser (Abbott Architect; Abbott Laboratories). The free androgen index (FAI) was calculated by the following equation: FAI = testosterone (nmol/L) × 100/SHBG (nmol/L) × 100 [23].

### 2.3. Euglycaemic-Hyperinsulinaemic Clamping (EHC)

EHC was used to as the gold standard to the diagnosis of IR and performed in all participants as previously reported [24]. Briefly, after fasting for 10-12 h, a catheter was placed in the antecubital vein to infuse insulin and glucose. Another catheter was placed retrograde in the dorsal vein of the contralateral hand for blood withdrawal. Regular human insulin (1 mU/kg minute) was infused for 2 h, and a variable infusion of 20% glucose was administered to maintain plasma glucose at the fasting level. During clamping, blood glucose levels were measured every 10-15 minutes to guide the glucose infusion. The rate of glucose disposal (GRd) was defined as the glucose infusion rate (GIR) during the stable period of the clamp and was related to body weight (*M*-value). Blood samples for ANGPTL8 measurements were obtained at 0, 80, 100, and 120 minutes. The samples were immediately cooled, and serum/plasma was prepared within 1 h and stored at -80°C until further use.

### 2.4. Cytokine Measurements

Serum ANGPTL8 concentration was determined with an ELISA obtained from Phoenix Pharmaceuticals Inc. (Belmont, CA, USA) by using the manufacturer's protocol. The intra-assay and interassay variations were <10% and <15%, respectively. The linear range of the assay was 0-100 *μ*g/L. The assay has high sensitivity and excellent specificity for the detection of ANGPTL8 with no significant cross-reactivity or interference. Serum ADI levels were also measured with an ELISA from Adipobiotech as previously described [25].

### 2.5. Statistical Analysis

All analyses were performed with Statistical Package for the Social Sciences version 19.0 (SPSS Inc., Chicago, IL). Normally distributed data were expressed as the mean ± SD. The data for nonnormal distribution were skewed and logarithmically transformed to obtain a normal distribution, which was expressed as the median with interquartile range (IQR). An unpaired *t* test or one-way ANOVA was performed to analyse the differences between two or more groups. Spearman's correlation analysis was used to examine the association of circulating ANGPTL8 with other parameters. Relationships between the ANGPTL8 and the other variables were investigated by using a multiple stepwise regression analysis, with ANGPTL8 as a dependent variable. A multivariate logistic regression analysis was used to investigate the association of ANGPTL8 with MetS. Receiver operating characteristic (ROC) curves were used to analyse the predictive values of serum ANGPTL8 for MetS and IR. All data were based on two-sided tests. *P* < 0.05 was considered statistically significant.

## 3. Results

### 3.1. Main Clinical Features, Hormone, and Serum ANGPTL8 Levels in Study Populations

**Figure 1:**
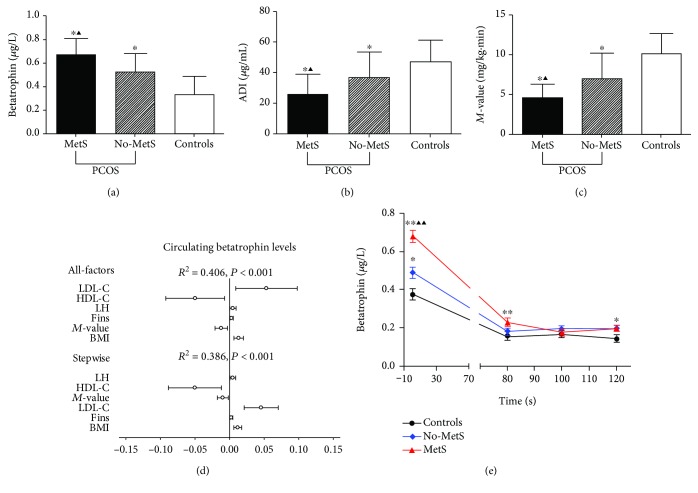
Parameters in PCOS women with MetS or without MetS and healthy controls. (a) Serum betatrophin levels in PCOS women with MetS were significantly higher than that of PCOS women without MetS and healthy controls (*P* < 0.05 or *P* < 0.01); (b) serum ADI concentrations, an adipocytokine-related insulin sensitivity, were markedly lower in MetS individuals than no-MetS individuals and healthy controls (*P* < 0.05 or *P* < 0.01); (c) PCOS women with MetS have lower *M*-values compare with PCOS women without MetS and/or healthy controls (*P* < 0.05 or *P* < 0.01); (d) in all study populations, regression analyses of all-factor and stepwise models indicated that the main predictors of circulating ANGPTL8 were LDL-C and BMI; (e) serum ANGPTL8 concentrations decreased significantly in all study individuals and in MetS individuals; circulating ANGPTL8 levels at the stable state of clamp were still significantly higher than that of healthy individuals.

The anthropometric and biochemical parameters in the study populations are shown in Table 1. PCOS women with MetS have higher BMI, Fat%, WHR, BP, TG, total cholesterol (TC), LDL-C, FBG, FIns, HbA1c, and HOMA-IR and lower HDL-C and *M*-values than PCOS women without MetS and/or healthy controls (*P* < 0.05 or *P* < 0.01; Table 1 and Figure 1(c)). Furthermore, in PCOS women with MetS, serum TEST, DHEAS levels, and the FAI were markedly higher, while PRL and SHBG was lower than those in PCOS women without MetS and/or healthy controls (*P* < 0.05 or *P* < 0.01; Table 1). Importantly, serum ANGPTL8 levels in PCOS women with MetS were significantly higher than those in PCOS women without MetS and healthy controls (*P* < 0.05 or *P* < 0.01; Table 1 and Figure 1(a)). Serum ANGPTL8 levels remained significantly different after adjustment for age and BMI (Table 1). Serum ADI concentrations, an adipocytokine-related insulin sensitivity, were markedly lower in MetS individuals than in non-MetS individuals and healthy controls (*P* < 0.05 or *P* < 0.01; Table 1 and Figure 1(b)).

### 3.2. The Association between Serum ANGPTL8 and Other Parameters in Study Populations

Spearman's correlation analysis showed that serum ANGPTL8 at baseline was correlated positively with age (*r* = 0.16, *P* < 0.01), BMI (*r* = 0.48, *P* < 0.001), FAT% (*r* = 0.43, *P* < 0.001), WHR (*r* = 0.43, *P* < 0.001), SBP (*r* = 0.20, *P* < 0.001), TG (*r* = 0.42, *P* < 0.001), FBG (*r* = 0.28, *P* < 0.001), HbA1c (*r* = 0.15, *P* < 0.05), FIns (*r* = 0.53, *P* < 0.001), and HOMA-IR (*r* = 0.53, *P* < 0.001) and negatively correlated with ADI (*r* = −0.44, *P* < 0.001) and *M*-values (*r* = −0.51, *P* < 0.001) in the study populations (Table 2). In addition, circulating ANGPTL8 was positively correlated with PRL (*r* = 0.17, *P* < 0.01), LH (*r* = 0.22, *P* < 0.001), TEST (*r* = 0.27, *P* < 0.001), and FAI (*r* = 0.34, *P* < 0.001) and negatively correlated with SHBG (*r* = −0.35, *P* < 0.001) in the study populations (Table 3). Furthermore, ADI was negatively correlated with BMI, FAT%, WHR, TG, FBG, FIns, HbA1c, HOMA-IR, LH, TEST, DHEAS, and FAI but positively correlated with *M*-value and SHBG (Tables 2 and 3). In all study populations, regression analyses of all-factor and stepwise models indicated that the main predictors of circulating ANGPTL8 were LDL-C and BMI (Figure 1(d)). The multiple regression equation was Y_ANGPTL8_ = 0.130 + 0.013X_BMI_ + 0.003X_FIns_ + 0.053 X_LDL‐C_‐0.012X_*M*‐value_‐0.050X_HDL‐C_ + 0.005X_LH_ (*R*^2^ = 0.406, *P* < 0.01).

In fully adjusted logistic regression models controlling for anthropometric variables, BP, lipid profile, and hormone, higher serum ANGPTL8 concentrations were markedly related to the high onset of MetS in individuals with PCOS (Table 4).

### 3.3. Effects of EHC on Circulating ANGPTL8 in the Study Populations

To investigate whether serum ANGPTL8 levels are affected by hyperinsulinaemia, EHC was performed in PCOS women with and without MetS and in healthy women. Insulin levels during EHC were elevated from 8.0 ± 3.3 to 110.1 ± 15.2 mU/L in healthy women and from 25.0 ± 6.0 to 105.2 ± 20.3 mU/L in PCOS women. Blood glucose was clamped at euglycaemic levels (~5.5 mmol/L) by an infusion of 25% glucose without significant hypoglycaemic events in these individuals. During EHC, *M*-values were markedly lower in MetS individuals than those in non-MetS and healthy women (Table 1), indicating more obvious IR in PCOS women with MetS. In response to hyperinsulinaemia, serum ANGPTL8 concentrations decreased significantly in all study individuals (Figure 1(e)). During the stable clamping state, circulating ANGPTL8 was maintained at a lower level in all three groups due to hyperinsulinaemia (from 0.38 ± 0.17 to 0.15 ± 0.09 *μ*g/L for the controls, from 0.49 ± 0.15 to 0.20 ± 0.08 *μ*g/L for non-MetS, and from 0.68 ± 0.14 to 0.20 ± 0.08 *μ*g/L for MetS). However, in MetS individuals, circulating ANGPTL8 levels in the stable clamping state were still significantly higher than those in healthy individuals (0.20 ± 0.08 vs. 0.15 ± 0.09 *μ*g/L, *P* < 0.05; Figure 1(e)). Therefore, in response to hyperinsulinaemia during EHC, circulating ANGPTL8 levels were significantly decreased. These results indicate that circulating ANGPTL8 is associated with dysmetabolism and IR.

### 3.4. The Predictive Value of Circulating ANGPTL8 in Detecting MetS and IR

**Figure 2:**
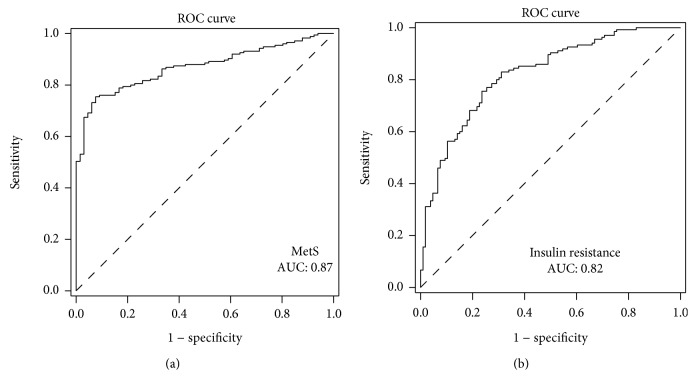
The ROC curve of circulating ANGPTL8 for predicting MetS and IR. (a) The area under the ROC curves was 0.87 (*P* < 0.001) with a sensitivity of 92.4% and specificity of 75.4% for MetS (AUC_MetS_); (b) the area under the ROC curves was 0.82 (*P* < 0.01) with a sensitivity of 76.4% and specificity of 75.6% for IR (AUC_IR_).

Finally, we performed the ROC curve of circulating ANGPTL8 for predicting MetS and IR. The results showed that the area under the ROC curves was 0.87 (*P* < 0.001) with a sensitivity of 92.4% and specificity of 75.4% for MetS (AUC_MetS_) (Figure 2(a)) and 0.82 (*P* < 0.01) with a sensitivity of 76.4% and specificity of 75.6% for IR (AUC_IR_) (Figure 2(b)). The best cutoff values for serum ANGPTL8 levels to predict MetS and IR were 0.53 and 0.51 *μ*g/L, respectively.

## 4. Discussion

MetS and PCOS have some similar clinical manifestations such as obesity and lipid metabolism disorders. The mechanism lies in the MetS, and PCOS is still unclear. Researchers hold there may have some correlation between MetS and PCOS that include: (1) insulin resistance is the milestone between MetS and PCOS; (2) some adipokines are associated with MetS and PCOS, such as leptin, insulin-like growth factor-1, and adiponectin. Insulin resistance is the common soil between MetS and PCOS, which has been generally recognized. Therefore, it is a promising work to find a specific target for regulating insulin resistance [3, 9, 13].

Although circulating ANGPTL8 levels have been reported to be related to IR [26], T2DM [27–30], obesity [27, 30], PCOS [31–33], nonalcoholic fatty liver disease (NAFLD) [34], and MetS [12, 35], ANGPTL8 is mainly secreted and expressed by hepatocytes. It reduces the cleavage of triglycerides by inhibiting the activity of lipoprotein esterase and increases the level of triglycerides. ANGPTL8 also promotes the proliferation of islet beta cells. Some researchers suggested that the serum ANGPTL8 level was significantly increased in type 2 diabetes patients; the ANGPTL8 level was positively correlated with insulin resistance and negatively correlated with insulin sensitivity [29]. High insulin level increases the ANGPTL8 expression by activating the PI3K/Akt pathway, while insulin resistance inhibits the ANGPTL8 expression [36]. Other researches declared that overexpression of ANGPTL8 may inhibit the PI3K/Akt pathway, reduce insulin sensitivity, and enhance insulin resistance in hepatocytes [31]. The results are inconsistent, and the regulation factors of ANGPTL8 are not clear. Therefore, as with most new discoveries, the association of circulating ANGPTL8 with these diseases needs to be studied repeatedly.

In the current study of PCOS and ANGPTL8, regardless of BMI, the circulatory ANGPTL8 levels are elevated in PCOS patients compared to controls. PCOS patients with higher insulin resistance had substantially higher circulating ANGPTL8 concentrations [37]. Other research suggested that ANGPTL8 levels were increased in women with PCOS and were associated with insulin resistance, hs-CRP, and free testosterone in these patients (Table 5). Elevated ANGPTL8 levels were found to increase the odds of having PCOS [38]. And one research declared that ANGPTL8 levels are reduced in full-blown PCOS patients and positively associated with low-density lipoprotein cholesterol [39]; therefore, further research is needed to elucidate the role of ANGPTL8 in PCOS and insulin resistance.

We investigated ANGPTL8 circulation levels in PCOS patients with or without MetS and in healthy women. The significance of this design is that we can exclude the effects of gender and age on the results. Our data showed that serum ANGPTL8 levels in healthy young women were lower than those in normal individuals, as reported by Abu-Farha et al. (0.33 ± 0.16 vs. 0.71 (0.59–1.15) *μ*g/L). In our study, MetS individuals had lower circulating ANGPTL8 levels than the MetS individuals reported by Abu-Farha et al. (0.67 ± 0.14 vs. 1.14 (0.17–1.17) *μ*g/L) [12]. In addition, circulating ANGPTL8 levels in our study were lower than those reported by Liu et al. (0.12 ± 0.08 *μ*g/L for MetS subjects and 0.13 ± 0.01 *μ*g/L for the controls) [35]. We consider that the difference between the present and previous studies may be due to the effects of age and sex on study populations. In the previous two studies, the study subjects were selected from individuals aged 18-70 years and with different sexes, while in our study, the age of the subjects was limited to 18-35 years, and only women were included in the study.

In the current study, we found that circulating ANGPTL8 levels were higher in PCOS women than in healthy women, and PCOS women with MetS also had higher circulating ANGPTL8 levels than PCOS women without MetS. These results suggest that with the aggravation of this metabolic disorder, the circulating levels of ANGPTL8 are progressively increased from normal young women to PCOS patients and then to PCOS patients with MetS. Therefore, ANGPTL8 may be a serum marker related to the degree of dysmetabolism *in vivo.*

It is noteworthy that our results were consistent with two published studies performed by Crujeiras et al. and Abu-Farha et al., in which circulating ANGPTL8 was increased in MetS individuals [12, 40]. However, in another study, there was no significant difference in circulating ANGPTL8 between the MetS and normal subjects [41]. This disparity may be due to confounding factors, such as anthropometric characteristics, age, and gender. All MetS individuals enrolled in this study were newly diagnosed without any drug treatment and were PCOS women aged 18-35 years and without T2DM.

In this study, we also found that fasting serum full-length ANGPTL8 levels were positively correlated with markers of adiposity (BMI, FAT%, and WHR), the glycometabolism index (FBF, 2 h-BG, and HbA1c), and IR markers (FIns and HOMA-IR) but negatively correlated with ADI and *M*-values. Consistent with the current results, previous reports from different groups also showed these correlations between ANGPTL8 and other parameters [28, 29]. In addition, our ROC curve analysis also indicated that circulating ANGPTL8 could predict MetS with a relatively high sensitivity and specificity. Therefore, the association between ANGPTL8 and glucose, adiposity, ADI, and IR parameters confirmed the potential role of ANGPTL8 in metabolic disorders and IR and thus contributed to the occurrence and development of MetS.

Our study had some strengths. First, ANGPTL8 levels were examined in young women; thus, miscellaneous factors in sex and age were excluded. Second, we used age- and gender-matched controls, making between group comparisons more feasible. Third, all individuals in this study were drug-naïve and untreated with diet control or exercise. Fourth, EHC, a gold standard for IR, was performed in all participants. Insulin sensitivity is accurately evaluated.

Our study has some limitations: (1) the design of this cross-sectional study cannot suggest causality, (2) our data could be affected by some outliers due to the related small sample size, and (3) circulating ANGPTL8 levels are based on single measurements, which may not reflect the alternations in ANGPTL8 levels over time. Serial alternations in circulating CTRP-5 concentrations should be measured at different stages of T2DM and IR to investigate the role of CTRP-5 in the development of T2DM. In addition, the study population consisted entirely of Chinese people. Thus, the application of these data to other ethnic populations should be undertaken with caution. Nevertheless, the use of newly diagnosed PCOS patients with and without MetS and their age- and gender-matched controls prevents pharmacotherapy complications or other confounding variables.

In conclusion, the current study shows that circulating ANGPTL8 concentrations are progressively increased from healthy controls to PCOS patients and then to PCOS patients with MetS. The high concentrations of ANGPTL8 in PCOS populations were related to the incidence of MetS. Our results highlight a possible role for ANGPTL8 in IR and MetS. In future studies, this cytokine might be used as a biomarker for MetS and IR.

## Figures and Tables

**Table 1:** Main clinical features and circulating betatrophin levels in study populations.

Group	PCOS		Controls	*P* value
	MetS	No-MetS		
*N*	65	78	98	—
Age (yr)^b^	26.2 ± 3.4	25.4 ± 4.9	25.7 ± 2.3	NS
BMI (kg/m^2^)^a^	25.9(23.2-30.4)^∗∗^^▲▲^	23.5(19.7-26.4)^∗∗^	20.0(18.6-21.3)	<0.001
FAT (%)	39.27±9.23^∗∗^^▲▲^	31.8±9.0^∗∗^	26.6 ± 5.5	<0.001
WHR^b^	0.89(0.83-0.93)^∗∗^^▲▲^	0.84(0.80-0.87)^∗∗^	0.78(0.75-0.84)	<0.001
SBP (mmHg)^b^	119(109-125)^∗∗^	114(107-120)^∗∗^	108(102-116)	<0.001
DBP (mmHg)	78 ± 9^∗^^▲^	75 ± 6	75 ± 8	<0.05
TG (mmol/L)^a^	2.30(1.66-3.08)^∗∗^^▲▲^	1.06(0.64-1.77)^∗^	0.80(0.59-1.29)	<0.001
TC (mmol/L)	4.64±0.95^∗∗^^▲^	4.24 ± 1.11^∗^	3.86 ± 1.00	<0.001
HDL-C (mmol/L)^b^	1.20(1.04-1.25)^∗∗^^▲▲^	1.41(1.19-1.58)^∗∗^	1.17(0.99-1.43)	<0.001
LDL-C (mmol/L)	2.69±0.86^∗∗^^▲^	2.34 ± 0.83	2.16 ± 0.87	<0.01
FFA (*μ*mol/L)	0.63 ± 0.18	0.55 ± 0.21	0.56 ± 0.28	NS
FBG (mmol/L)^a^	5.15(4.74-5.76)^∗∗^^▲▲^	4.81(4.43-5.05)^∗∗^	4.42(4.03-4.74)	<0.001
FIns (pmol/L)^b^	19.24 (13.3-28.3)^∗∗^^▲▲^	9.59(6.80-17.75)^∗∗^	7.02(6.10-8.45)	<0.001
HbA1c (%)^b^	5.30(5.10-5.65)^∗∗^	5.30(5.10-5.50)^∗∗^	5.20(5.00-5.30)	<0.001
HOMA-IR^b^	4.55(2.68-6.34)^∗∗^^▲▲^	2.05(1.52-3.55)^∗∗^	1.35(1.16-1.73)	<0.001
M-value (mg/kg/min)^a^	4.12(30.48-5.71)^∗∗^^▲▲^	5.91(4.95-8.77)^∗∗^	10.29(8.07-11.94)	<0.001
ADI (*μ*g/mL)	26.4±12.6^∗∗^^▲▲^	36.5±16.9^∗∗^	47.06 ± 14.04	<0.001
ANGPTL8 (*μ*g/L)	0.67±0.14^∗∗^^▲▲^	0.52±0.16^∗∗^	0.33 ± 0.16	<0.001
PRL (mIU/L)^b^	337.1(230.8-561.6)^∗∗^	381.6(216.8-490.0)	374.6(235.0-404.6)	NS
PROG (nmol/L)^b^	2.81(1.87-3.12)	2.81(2.18-3.12)	2.50(1.87-3.20)	NS
LH (IU/L)^a^	7.32(4.56-11.90)^∗∗^	10.80(6.21-15.09)^∗∗^	4.31(3.04-6.16)	<0.001
FSH (IU/L)^a^	7.60(6.10-8.63)	7.42(6.18-9.10)	7.80(6.73-9.26)	NS
TEST (nmol/L)^b^	2.99(1.95-3.86)^∗∗^	2.79(2.20-3.43)^∗∗^	1.65(1.21-2.29)	NS
E2 (pmol/L)^b^	212.9(126.6-280.8)	194.5(98.4-244.1)	183.5(119.6-255.1)	NS
DHEAS (*μ*g/dL)^b^	202.9(163.3-149.5)^∗^	183.4.0(150.4-214.3)	182.1(141.9-217.0)	<0.05
SHBG (nmol/L)^a^	30.6(17.8-42.2)^∗∗^^▲▲^	40.6(24.2-75.2)^∗∗^	57.4(42.0-75.6)	<0.001
FAI^a^	9.45(4.83-16.96)^∗∗^^▲^	6.78(3.47-10.21)^∗∗^	2.59(1.80-4.97)	<0.001

BMI: body mass index; FAT%: body fat %; WHR: waist-to-hip ratio; SBP: systolic blood pressure; DBP: diastolic blood pressure; TG: triglyceride; TC: total cholesterol; HDL-C: high-density lipoprotein cholesterol; LDL-C: low-density lipoprotein cholesterol; FFA: free fatty acid; FBG: fasting blood glucose; FIns: fasting insulin; HOMA-IR: HOMA-insulin resistance index; *M*-value: whole body glucose uptake rate; ADI: adiponectin; PRL: prolactin; PROG: progestogen; LH: luteinizing hormone; FSH: follicle-stimulating hormone; TEST: total testosterone; E2: estradiol; DHEAS: dehydroepiandrosterone sulfate; SHBG: sex hormone-binding globulin. FAI: free androgen index. Data are median (interquartile range) or frequency (percent). ^a^Log transformed before analysis; ^b^nonparametric test was used in comparisons between those groups. ^∗^*P* < 0.05, compared with controls; ^▲^*P* < 0.05, compared with no MetS; ^∗∗^*P* < 0.01, compared with the controls; ^▲▲^*P* < 0.01, compared with no MetS.

**Table 2:** Spearman's correlation coefficients between circulating ANGPTL8 and other parameters.

Group	ANGPTL8	ADI	*M*-value	Age	BMI	FAT (%)	WHR	SBP	DBP	TG	HDL-C	FBG	FIns	HbA1c	HOMA-IR
ANGPTL8	1	-0.440 ^a^	-0.507^a^	0.159^b^	0.475^a^	0.433^a^	0.425^a^	0.202^a^	0.065	0.424^a^	-0.003	0.280^a^	0.527^a^	0.147^c^	0.533^a^
ADI		1	0.605^a^	-0.127	-0.479^a^	-0.453^a^	-0.387^a^	-0.138	0.065	-0.299^a^	0.056	-0.272^a^	-0.475^a^	-0.244^a^	-0.483^a^
*M*-value			1	-0.110	-0.599^a^	-0.708^a^	-0.495^a^	-0.270^a^	-0.100	-0.460^a^	-0.028	-0.360^a^	-0.701^a^	-0.368^a^	-0.0709
Age				1	0.160^b^	0.168^a^	0.154^c^	0.50	0.106	0.074	0.064	0.167^a^	0.063	-0.013	0.098
BMI					1	0.771^a^	0.517^a^	0.288^a^	0.130^c^	0.368^a^	0.041	0.247^a^	0.547^a^	0.094	0.555^a^
FAT (%)						1	0.551^a^	0.358^a^	0.167^a^	0.409^a^	-0.009	0.383^a^	0.645^a^	0.148^c^	0.655^a^
WHR							1	0.267^a^	0.101	0.292^a^	-0.043	0.167^a^	0.456^a^	0.204^a^	0.448^a^
SBP								1	0.417^a^	0.071	-0.066	0.111	0.223^a^	0.120	0.224^a^
DBP									1	-0.069	-0.022	-0.008	0.025	0.039	0.020
TG										1	-0.038	0.289^a^	0.392^a^	0.182^a^	0.415^a^
HDL-C											1	0.039	-0.015	0.008	-0.003
FBG												1	0.410^a^	0.179^a^	0.555^a^
FIns													1	0.249^a^	0.981^a^
HbA1c (%)														1	0.255^a^
HOMA-_IR_															1

a: *P* < 0.001; b: *P* < 0.01; c: *P* < 0.05.

**Table 3:** Spearman's correlation coefficients between ANGPTL8 and sex hormone.

Group	ANGPTL8	PRL	PROG	LH	FSH	TEST	E2	DHEAS	SHBG	FAI
ANGPTL8	1	0.167^b^	-0.078	0.219^a^	-0.055	0.271^a^	0.070	0.117	-0.349^a^	0.342^a^
*M*-value		-0.172 ^a^	0.024	-0.199^a^	0.099	-0.314^a^	-0.024	-0.205^a^	0.494^a^	-0.466^a^
ADI		-0.101	0.048	-0.204^a^	0.096	-0.291^a^	0.022	-0.207^a^	0.392^a^	-0.416^a^
PRL		1	0.007	0.024	0.020	0.201^a^	0.145^c^	0.027	-0.274^a^	0.249^a^
PROG			1	-0.039	-0.119	-0.003	0.055	0.055	0.055	-0.081
LH				1	0.115	0.457^a^	0.018	-0.060	-0.299^a^	0.418^a^
FSH					1	0.008	-0.050	-0.051	-0.008	0.028
TEST						1	0.191^a^	0.284^a^	-0.361^a^	0.814^a^
E2							1	0.152^c^	-0.191^a^	0.199^a^
DHEAS								1	-0.329^a^	0.347^a^
SHBG									1	-0.798^a^
FAI										1

a: *P* < 0.001; b: *P* < 0.01.

**Table 4:** Association of circulating ANGPTL8 with MetS in fully adjusted models.

Model adjust	MetS			Insulin resistance		
	OR	95% CI	*P*	OR	95% CI	*P*
Age	6.69	3.90-11.47	<0.001	4.82	3.17-7.32	<0.001
Age, BMI	4.79	2.74-8.36	<0.001	3.19	2.07-4.93	<0.001
Age, BMI, WHR	4.47	2.49-8.03	<0.001	3.27	2.11-5.08	<0.001
Age, BMI, WHR, HbA1c	4.51	2.49-8.17	<0.001	3.24	2.06-5.12	<0.001
Age, BMI, WHR, HbA1c, FIns	3.95	2.15-7.26	<0.001	2.19	1.29-3.71	<0.01
Age, BMI, WHR, HbA1c, FIns, lipid profile	4.12	2.16-7.87	<0.001	2.49	1.42-4.36	<0.01
Age, BMI, WHR, HbA1c, FIns, lipid profile, hormone	5.71	2.51-13.0	<0.001	2.43	1.29-4.61	<0.01

Results of binary logistic regression analysis are presented as the odds ratio (OR) of being in MetS status decrease in circulating. BMI: body mass index; WHR: waist-to-hip ratio; FAT (%): the percentage of fat *in vivo*; SBP: systolic blood pressure; DBP: diastolic blood pressure; lipid profile: including total cholesterol, FFA, triglyceride, and LDL- and HDL-cholesterol. Hormone: including SHBG, DHEAS, E2, TEST, LH, FSH, PRL, and PROG.

**Table 5:** Row mean scores and Cochran-Armitage trend test of the impact of circulating ANGPTL8 levels on MetS.

	MetS	
	*x* ^2^	*P* value
Row mean scores test	76.8259	<0.001
Cochran-Armitage trend test	5.5862	<0.001

The circulating ANGPTL8 levels of all subjects were cut-off and adjusted for age, sex, BMI, WHR, BP, and lipid profile.

## Data Availability

The individual unidentified participant data (including data dictionaries), study protocol, and statistical analysis plan will be available from the corresponding author upon request.

## Abbreviations

PCOS: Polycystic ovary syndrome
BMI: Body mass index
FAT%: Body fat %
WHR: Waist-to-hip ratio
SBP: Systolic blood pressure
TG: Triglyceride
FBG: Fasting blood glucose
FIns: Fasting insulin
HOMA-IR: HOMA-insulin resistance index
PRL: Prolactin
LH: Luteinizing hormone
TEST: Total testosterone
FAI: Free androgen index
SHBG: Sex hormone-binding globulin
AUC: The area under the receiver operating characteristic (ROC) curve.
